# Supplementary material for: Identification of natural products and synthetic analogs which inhibit microsporidia spores and prevent infection
Source: bioRxiv. 2025 Aug 6:2025.08.06.669004. Preprint. [Version 1] doi: 10.1101/2025.08.06.669004 (PMC12340860; doi:10.1101/2025.08.06.669004)
Supplement: Supplement 2 [file NIHPP2025.08.06.669004v1-supplement-2.pdf]

bioRxiv preprint doi: https://doi.org/10.1101/2025.08.06.669004; this version posted August 6, 2025. The copyright holder for this preprint (which was not certified by peer review) is the author/funder, who has granted bioRxiv a license to display the preprint in perpetuity. It is made available under aCC-BY 4.0 International license.

Supplementary materials

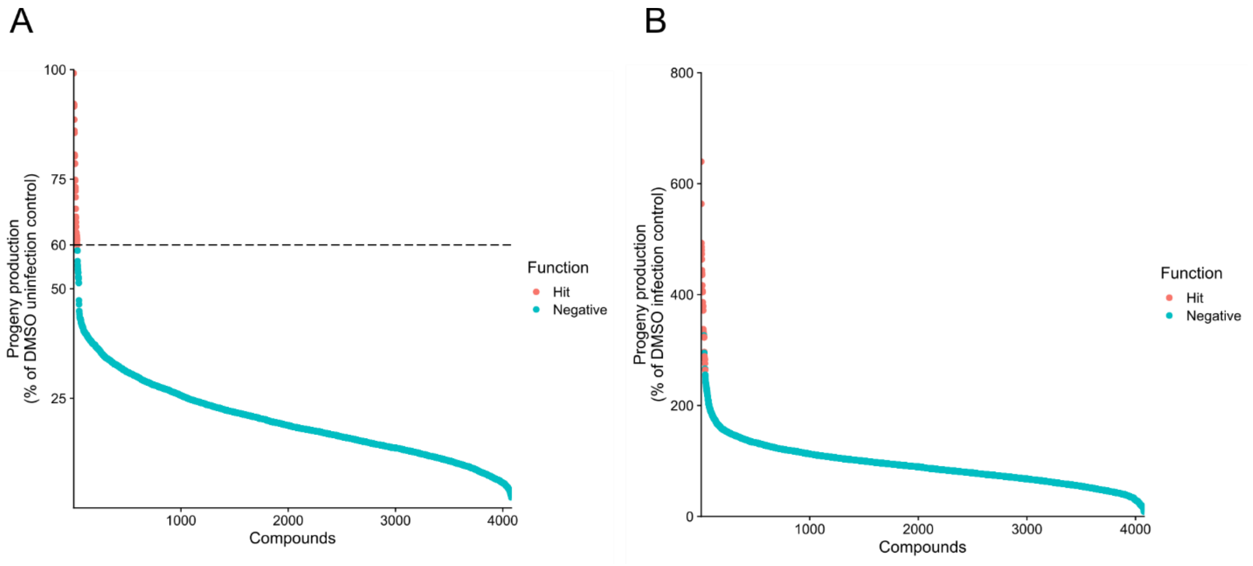

Fig. S1. Ranked progeny production and fold increase for 4,080 BU-CMD compounds tested against *N. parisii* infected *C. elegans*. (A) Data described in figure 1A, which is the percentage of progeny production compared to uninfected controls, is presented in ranked order. **(B)** The percentage of progeny production compared to infected controls. In both (A) and (B) the compounds are colored as in Figure 1A with compounds with progeny production less than 60% are shown colored blue and compounds with progeny production of at least 60% are shown colored red.

bioRxiv preprint doi: https://doi.org/10.1101/2025.08.06.669004; this version posted August 6, 2025. The copyright holder for this preprint (which was not certified by peer review) is the author/funder, who has granted bioRxiv a license to display the preprint in perpetuity. It is made available under aCC-BY 4.0 International license.

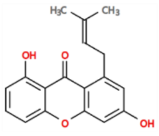
CMLD012462

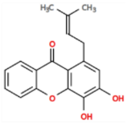
CMLD013363

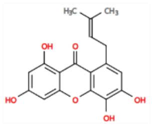
CMLD013228

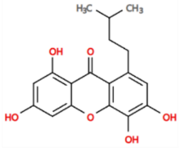
CMLD012383

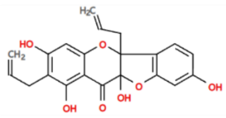
CMLD010544

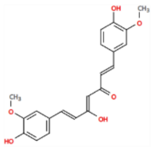
CMLD011737

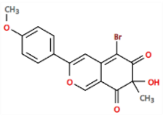
CMLD003390

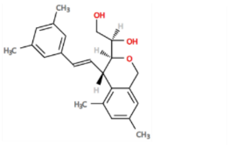
CMLD006241

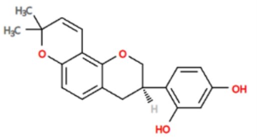
CMLD013691

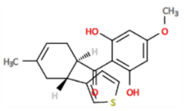
CMLD011181

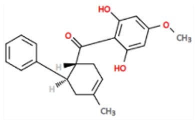
CMLD011395

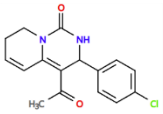
CMLD011363

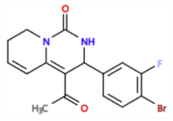
CMLD011595

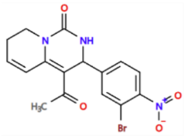
CMLD013534

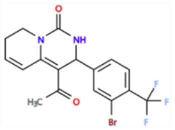
CMLD013532

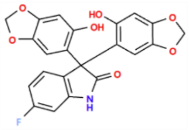
CMLD012338

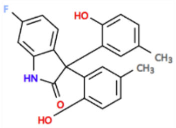
CMLD013615

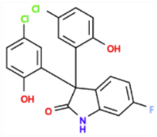
CMLD013616

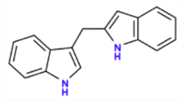
CMLD012632

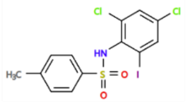
CMDNG0774

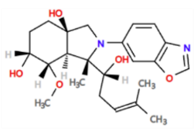
CMLD008987

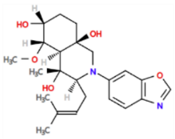
CMLD008995

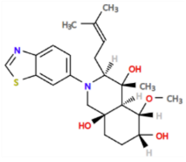
CMLD008983

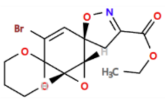
CMLD011481

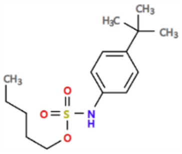
CMDJR0075

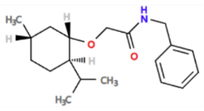
CMDNG0014

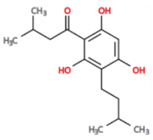
CMLD011770

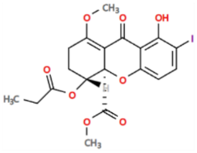
CMLD010971

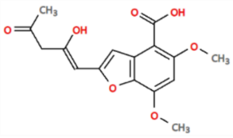
CMLD011778

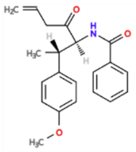
CMDJJ0030

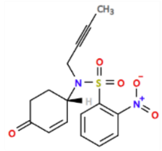
CMDNG0445

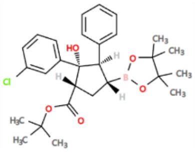
CMDJJ0077

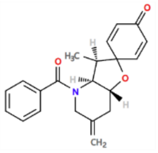
CMDJJ0040

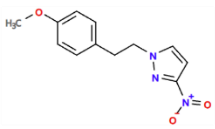
CMLD012930

Figure S2. Chemical structures of the 34 BU-CMD compounds with progeny production of at least 60%. The structure of each compound is shown with the label below the compound. Compounds are ordered the same as in Figure 1B.

bioRxiv preprint doi: https://doi.org/10.1101/2025.08.06.669004; this version posted August 6, 2025. The copyright holder for this preprint (which was not certified by peer review) is the author/funder, who has granted bioRxiv a license to display the preprint in perpetuity. It is made available under aCC-BY 4.0 International license.

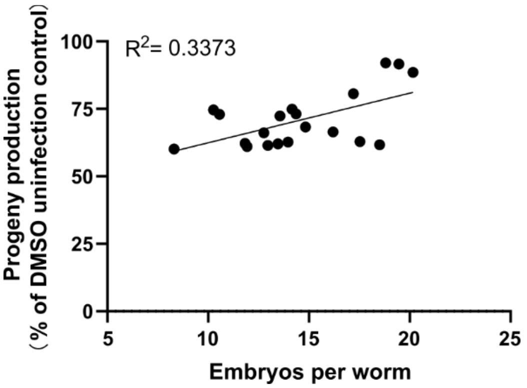

Figure S3. Progeny production from a screen of BU-CMD compounds is modestly correlated with number of embryos per worm from validation experiments. A linear correlation was performed between the percentage progeny production from Figure 1A and the number of embryos per worm for the 20 BU-CMD compounds tested in Figure 2A.

bioRxiv preprint doi: https://doi.org/10.1101/2025.08.06.669004; this version posted August 6, 2025. The copyright holder for this preprint (which was not certified by peer review) is the author/funder, who has granted bioRxiv a license to display the preprint in perpetuity. It is made available under aCC-BY 4.0 International license.

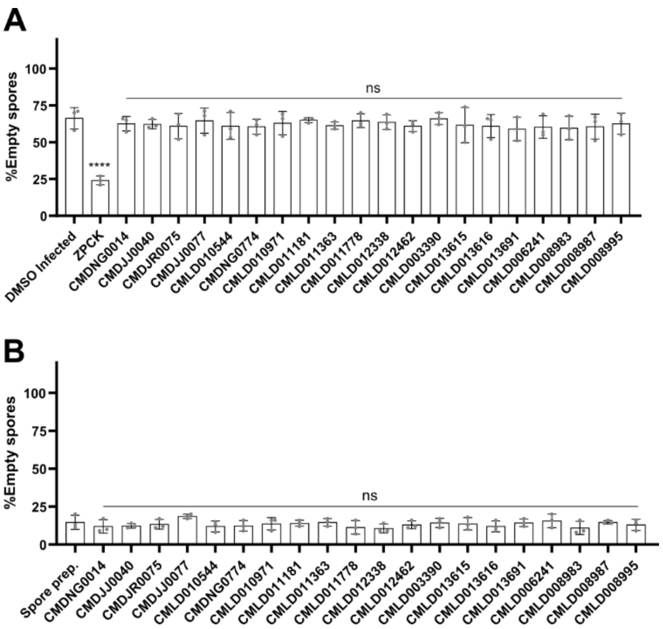

Figure S4. Validated BU-CMD compounds do not prevent spore germination in vivo nor trigger sporing firing *in vitro*. (A) *N. parisii* spores were incubated with compounds for 24 hours and the compounds were washed away from the spores. These spores were then incubated with L1 stage worms for 3 hours, and then worms were fixed and stained with FISH probe specific to the *N. parisii* 18S rRNA and DY96. Percentage of spores not containing a sporoplasm was quantified. **(B)** *N. parisii* spores were incubated with compounds for 24 hours and the compounds were washed away from the spores. Spores were then fixed and stained with FISH probe specific to the *N. parisii* 18S rRNA and DY96. Percentage of empty spores (spores not containing a sporoplasm). n = 3, N = ≥ 100 spores counted per biological replicate. The P-values were determined by one-way ANOVA with post hoc test (***$p < 0.001$ and ns means not significant). Means ± SD (horizontal bars) are shown.
